# Successful resection of rectal cancer and perirectal abscess following systemic chemotherapy and chemoradiotherapy: A case report

**DOI:** 10.1016/j.ijscr.2023.108403

**Published:** 2023-06-15

**Authors:** Takuya Yano, Kanyu Nakano, Masanori Yoshimitsu, Hitoshi Idani, Masazumi Okajima

**Affiliations:** a Department of Surgery, Hiroshima City Hiroshima Citizens Hospital, 7-33 Motomachi, Naka-ku, Hiroshima 730-8518, Japan; b Department of Gastroenterological and Transplant Surgery, Applied Life Sciences, Institute of Biomedical & Health Sciences, Hiroshima University, 1-2-3 Kasumi, Minami-ku, Hiroshima 734-8551, Japan

**Keywords:** Perirectal abscess, Rectal cancer, Total neoadjuvant therapy

## Abstract

**Introduction and importance:**

Perirectal abscesses are uncommon in colorectal cancer. Although abscess infection should be controlled before colorectal cancer treatment, abscess formation makes surgical resection and preoperative treatment difficult. There is currently no established treatment for colorectal cancer with perirectal abscesses. Here, we present a case of rectal cancer with a perirectal abscess that was resected after systemic chemotherapy followed by chemoradiotherapy.

**Case presentation:**

A 73-year-old man presented to the outpatient clinic with complaints of weight loss and general malaise. Colonoscopy revealed a circumferential tumor 3 cm from the anal verge, and examination of the endoscopic biopsy specimen indicated a well-differentiated tubular adenocarcinoma. Pelvic magnetic resonance imaging revealed a perirectal abscess on the ventral aspect of the rectum. After sigmoid colostomy was performed to control the infection, 4 cycles of panitumumab and modified fluorouracil, leucovorin, and oxaliplatin were administered. After the perirectal abscess disappeared, chemoradiotherapy to the whole pelvis (radiotherapy 45Gy/25 fractions plus tegafur-gimeracil-oteracil) was administered. Total pelvic exenteration with an ileal conduit was performed via open surgery. The pathological diagnosis was well-differentiated tubular adenocarcinoma with complete resection and negative resection margins. No recurrence of cancer has been observed 26 months after surgery.

**Clinical discussion:**

Treatment of colorectal cancer with perirectal abscess is difficult to define the extent of resection due to the spread of inflammation. We believe that treatment should address high risk of local recurrence.

**Conclusion:**

After sigmoid colostomy, complete resection of colorectal cancer with perirectal abscess could be achieved by systemic chemotherapy followed by chemoradiotherapy.

## Highlights

• Perirectal abscesses with colorectal cancer should be controlled before colorectal cancer treatment, abscess formation increases the risk of recurrence and makes surgical resection and preoperative treatment difficult.
• After controlling the abscess by sigmoid colostomy, systemic chemotherapy followed by chemoradiotherapy was considered to be a useful treatment because it achieved complete resection of colorectal cancer with perirectal abscess.

## 1 Introduction

The number of patients with advanced colorectal cancer and obstructive colorectal cancer is increasing [1]. Colorectal cancer with perirectal abscess is uncommon [2,3]. The mechanism by which rectal cancer is associated with abscesses is unknown. Infection of abscesses associated with colorectal cancer should be controlled before or during colorectal cancer treatment. However, it can be difficult to control the infection and perform curative surgery because it is necessary to consider changes in the surrounding tissues due to inflammation. Furthermore, rectal cancer complicated by perirectal abscess may be difficult to distinguish from the spread of cancer and inflammation, and the possibility of local recurrence after surgery is high, making curative surgery difficult to perform. There is currently no established treatment method for colorectal cancer associated with perirectal abscesses. Here, we present a case of rectal cancer with a perirectal abscess that was resected after systemic chemotherapy followed by chemoradiotherapy (CRT). This study was conducted according to the SCARE guidelines [4].

## 2 Presentation of case

**Fig. 1:**
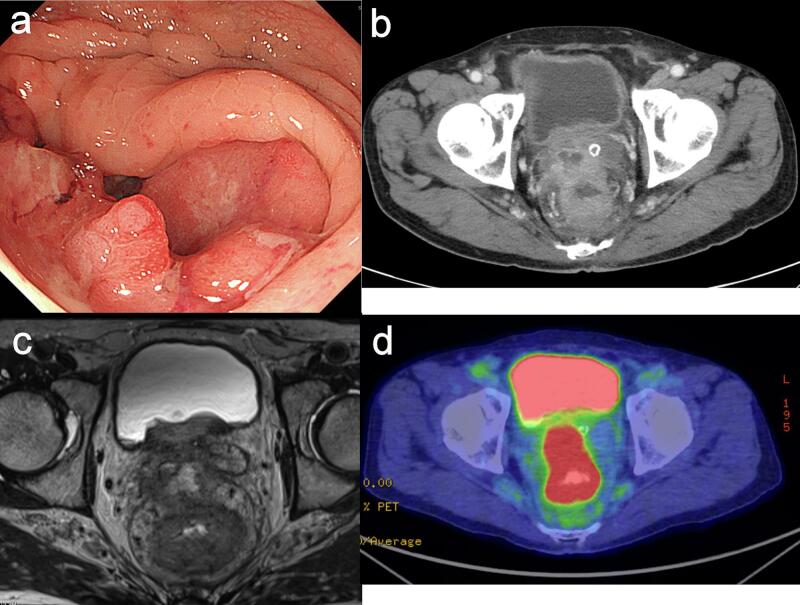
Radiological and colonoscopy findings before colostomy construction. A) Colonoscopy showing a circumferential ulcerated type tumor in the lower to upper right rectal wall. B) Abdominal contrast-enhanced computed tomography scan showing enhanced wall thickness and a lesion in the rectum, invasion of the bladder and prostate, and an abscess cavity on the ventral side of the rectum. C) Pelvic magnetic resonance imaging showing an abscess cavity on the ventral side of the rectum, bordering the bladder and prostate. D) ^18^F-fluorodeoxyglucose (FDG)-positron emission tomography/computed tomography showing FDG uptake in the rectal tumor and abscess.

A 73-year-old man was admitted to our hospital with complaints of weight loss and general malaise. A mass was palpated 3 cm from the anal verge on digital rectal examination. The patient was referred to our department for further examination and treatment. His medical history included gastric ulcer. He had no significant family history. Laboratory tests showed CEA 4.8 ng/mL (normal value <5.0 ng/mL) and CA19–9 18.8 U/mL (normal value <37.0 U/mL). Colonoscopy revealed a circumferential ulcerated type tumor with clear margin in the lower to upper rectum, and pathological examination of the endoscopic biopsy specimen indicated a well-differentiated tubular adenocarcinoma (Fig. 1A). Abdominal contrast-enhanced computed tomography (CT) showed rectal wall thickening, potential metastasis in the pericolic, bilateral internal iliac, and para-aortic lymph nodes, and invasion of the bladder and prostate (Fig. 1B). Pelvic magnetic resonance imaging (MRI) revealed a 23 × 22 mm perirectal abscess on the ventral side of the rectum (Fig. 1C). MRI TNM staging of the rectal cancer was T4b N1b M1a stageIVA. Because the patient had both rectal cancer and a perirectal abscess, sigmoid colostomy was performed. The para-aortic lymph nodes were sampled, and metastasis was not detected pathologically. There was the abscess infiltrating the prostate, and considering the risk of bleeding, we did not attempt interventional radiologic drainage. No antibiotics were administered, and clinically the infection caused by the perirectal abscess improved. 18F-fluorodeoxyglucose (FDG)-positron emission tomography/computed tomography revealed FDG uptake in the rectal tumor and abscess (Fig. 1D). Furthermore, genetic tests diagnosed the expression of KRAS wild-type and BRAFV600E wild-type using the endoscopic biopsy specimen.

**Fig. 2:**
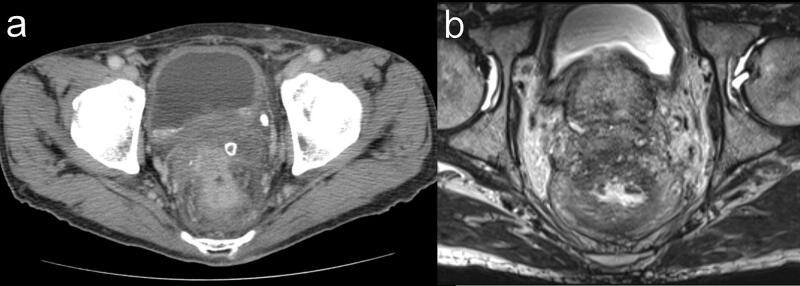
Radiological findings after 4 cycles of panitumumab and mFOLFOX6. A) Abdominal contrast-enhanced computed tomography showing reduced rectal wall thickness. B) Pelvic magnetic resonance imaging showing no indication of the perirectal abscess. mFOLFOX6, modified fluorouracil, leucovorin, and oxaliplatin.

**Fig. 3:**
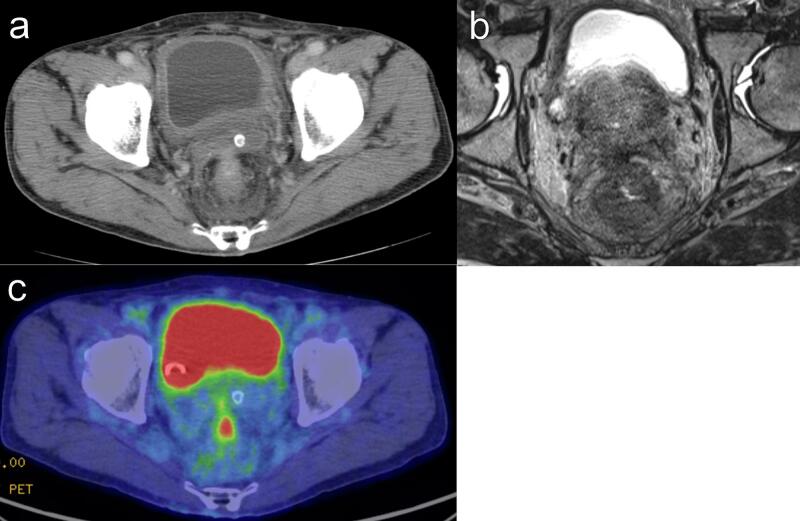
Radiological and colonoscopy findings before total pelvic exenteration. A) Abdominal contrast-enhanced computed tomography showing significant reduction in rectal wall thickness. B) Pelvic magnetic resonance imaging showing no perirectal abscess and an indistinct border between the tumor and the prostate. C) ^18^F-fluorodeoxyglucose (FDG)-positron emission tomography/computed tomography showing a reduction in FDG uptake and absence of distant metastasis.

**Fig. 4:**
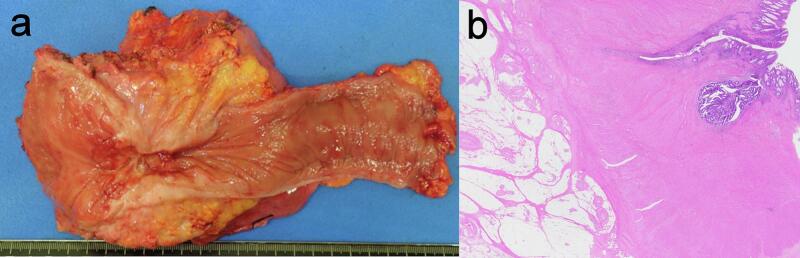
Pathological findings. Total pelvic exenteration with ileal conduit was performed to obtain a negative resection margin. A) Macroscopic findings of the resected specimen. B) Histological examination of the resected specimen indicated well-differentiated adenocarcinoma with massive necrotic tissue. The margins were negative.

Forty-two days after colostomy, administration of panitumumab and modified fluorouracil, leucovorin, and oxaliplatin was started, and the patient received 4 cycles. The perirectal abscess disappeared after chemotherapy in CT and MRI scan (Fig. 2A-B). Because MRI showed T4bN1aM0 stageIIIC, which was also suspected to involve the prostate, CRT to the whole pelvis (radiotherapy [RT] of 45 Gy/25 fractions plus tegafur-gimeracil-oteracil) was administered. CT, pelvic MRI, and colonoscopy showed significant reduction of the tumor. CT and MRI scans showed an indistinct border between the tumor and the prostate (Fig. 3A-B). MRI TNM staging of the rectal cancer was T4b N1a M0 stageIIIC on preoperative scan. ^18^F-fluorodeoxyglucose-positron emission tomography/CT showed reduced uptake in the rectum and no distant metastases (Fig. 3C). Fifty-seven days after the completion of CRT, total pelvic exenteration with an ileal conduit was performed by open surgery (Fig. 4A). The rectum and bladder could not be dissected at all because the border between the rectum and bladder could not be identified due to inflammation or cancerous infiltration. The dorsal side of the rectum could be dissected despite the influence of inflammation. Both sides of the rectum could also be dissected by combined resection of the pelvic plexus. The total pelvic exenteration was performed because dissection of the rectum, bladder and prostate would have cut into the cancerous tissue and potentially spread tumor cells. As a result, a tight surgical margin that would have required intraoperative frozen section biopsy could have been avoided. The pathological diagnosis was well-differentiated tubular adenocarcinoma without lymph node metastases or tumor cells in the radial margin. The assessment of the response to chemotherapy/RT was grade 2 (moderate effect) (Fig. 4B). Grade 2 is defined as follows: Prominent tumor cell necrosis, degeneration, lytic change, and/or disappearance is present in more than two-thirds of the entire lesion, but viable tumor cells remain [5]. The final pathological stage was ypT2(MP), ypN0, M0, ypStage I according to the Japanese Classification of Colorectal, Appendiceal, and Anal Carcinoma, 9th edition [5]. Finally, 15 days after postoperative hospitalization due to grade 2 paralytic ileus and urinary infection [6], the patient was discharged and received regular follow-up at the outpatient department for 26 months without recurrence.

## 3 Discussion

Abscess formation associated with colorectal cancer is uncommon and is observed in 0.3 %–4 % of colorectal cancer cases [2,3]. Rectal cancer with abscess formation is difficult to diagnose. This leads to delays in diagnosis and management, resulting in high morbidity and mortality rates. The optimal treatment for advanced rectal cancer with abscess formation has not been established. In this case, complete resection was achieved using multimodal treatment.

The following three clinical classifications of perforation-complicated colorectal cancer have been proposed: 1) free perforation with leakage of bowel contents into the peritoneal cavity, 2) covered perforation with local abscess formation, and 3) perforation into one of the neighboring organs or fistula formation [2]. However, the mechanism underlying abscess formation associated with colorectal cancer remains unknown.

After tumor perforation, tumor cells may spread transcoelomically, which has been shown to increase local recurrence and decrease survival [7]. Okita et al. reported that one of three cases of colorectal cancer complicated by an abscess had disseminated recurrence [8]. Further, it is difficult to distinguish between cancer and inflammation during infection because of the spread of inflammation caused by the abscess. Therefore, it is often difficult to set the resection line. Preoperative treatment is sometimes attempted to resolve this issue; however, no treatment method has been established. Surgery may be curative in patients with localized perforation of rectal carcinoma [7]. Complete resection of the colon cancer and the abscess wall is desirable; however, patients are often in poor general condition. Therefore, it is necessary to decide whether to perform minimal procedures and postpone major procedures until the patient's condition improves, or whether to perform major procedures immediately, taking into account the surgical risks [2]. Here, we selected colostomy to control the infection. In this case, preoperative imaging and intraoperative findings failed to confirm the boundary between the rectum, bladder and prostate, therefore the total pelvic exenteration was ultimately performed. Pathological examination revealed that the tumor was confined in the muscularis propria. Importantly, although the rectum and bladder could not be dissected due to inflammation caused by the abscess, and it was difficult to identify the extent of cancer invasion intraoperatively, adequate preoperative treatment had made resection of the cancer possible.

Michowitz et al. reported surgical mortality and 5-year survival rates of 50 % and 20 %, respectively, in patients with colon cancer complicated by local abscess [2]. A few cases of advanced rectal cancer complicated by infection were reported to result in Fournier's syndrome [9]. Because of the high mortality rate of Fournier's syndrome, it is necessary to control the infection well before preoperative treatment is initiated. Therefore, it is desirable to establish a treatment for rectal cancer with abscess formation because of the high risk of surgical complications and postoperative local recurrence.

Induction or consolidation chemotherapy with CRT before surgery for locally advanced rectal cancer, referred to as total neoadjuvant therapy (TNT), has been reported by several centers. Accumulating evidence shows that TNT in patients with locally advanced rectal cancer is expected to improve survival and reduce distant metastasis via systematic chemotherapy to prevent the onset of micrometastases [10]. Early results have demonstrated high rates of completion and tolerability, with complete pathological response rates between 20 % and 30 %. This allows more patients to undergo sphincter-preserving surgery, R0 resection or organ preservation management [11,12]. TNT is being tested in clinical trials for advanced rectal cancer; however, there is no established protocol, and it is not clear whether induction or consolidation chemotherapy is preferable. CRT for rectal cancer has been reported to cause perforation, although this is rare [[13], [14], [15], [16], [17]]. Some studies have reported an increased risk of pelvic septic complications after preoperative RT [[16], [17], [18]]. CRT may aggravate infection when abscesses are present and may not be fully effective when patients have increased inflammation. Gordeyev, S et al. reported that the presence of perirectal abscesses and fistulas in patients with locally advanced rectal cancer were not associated with increased toxicity or inferior clinical outcomes after preoperative CRT [19]. However, 22 of the 33 patients (66.7 %) had a colostomy created prior to CRT to control infection before CRT. Sato et al. reported that 1 of 14 patients died of pelvic abscesses after CRT [20]. RT may exacerbate and prolong infection, and RT in patients with colorectal cancer complicated by abscesses should be performed with caution. Therefore, when performing TNT for rectal cancer with abscess formation, consolidation therapy, which consists of preoperative chemotherapy followed by RT, may be an option after controlling the infection.

## 4 Conclusions

When preoperative treatment is performed for colorectal cancer with abscess formation, complications associated with the preoperative treatment must be considered. However, few cases of colorectal cancer with abscess formation have been reported, and it is difficult to organize clinical trials. Therefore, we believe that this case of complete resection with preoperative multimodality treatment provides valuable insights on the subject.

## Ethical approval

The case study was approved by the institutional review committee of Hiroshima City Hiroshima Citizens Hospital (2023-50). Written informed consent was obtained from the patient for publication of this case report.

## Sources of funding

This research received no specific grants from any funding agency in the public, commercial, or not-for-profit sectors.

## Authors' contributions

TY drafted the manuscript and treated the patient. KN treated the patient and helped draft the manuscript. MY, HI and MO determined the treatment plan and revised the manuscript. All the authors have read and approved the final manuscript.

## Guarantor

Takuya Yano, the corresponding author of this manuscript accept full responsibility for the work and the conduct of the study, access to the data and controlled the decision to publish.

## Registration of research studies

Not applicable. This case report involved no patient recruitment.

## Patient consent

Written informed consent was obtained from the patient for publication of this case report and accompanying images. A copy of the written consent form is available for review.

## Declaration of competing interest

The authors declare that they have no competing interests.

## Data Availability

The data supporting the findings of this study are available from the corresponding author, TY, upon reasonable request.
